# Enhancing Bone Regeneration Using Blended Poly(L-lactide-co-D, L-lactide) and β-Tricalcium Phosphate Nanofibrous Periodontal Biodegradable Membranes

**DOI:** 10.3390/polym17030256

**Published:** 2025-01-21

**Authors:** Princess Joy Naig, Zih-Yin Kuo, Min-Fan Chung, Chih-Hao Chen, Chi-Yun Wang, Kuo-Yung Hung

**Affiliations:** 1 Department of Biomedical Engineering and Medical Devices, Ming Chi University of Technology, New Taipei City 24301, Taiwanchiyunw@mail.mcut.edu.tw (C.-Y.W.); 2 Research Center for Intelligent Medical Devices, Ming Chi University of Technology, New Taipei City 24301, Taiwan; zihyin@mail.mcut.edu.tw; 3 NAN YA Plastics Corporation, Plastics 1st Division, New Taipei City 238, Taiwan; 4 Department of Plastic and Reconstructive Surgery, Chang Gung Memorial Hospital at Keelung, Keelung 204, Taiwan; 5 Department of Plastic and Reconstructive Surgery, Chang Gung Memorial Hospital at Linkou, Chang Gung University, College of Medicine, Taoyuan 333, Taiwan; 6 Bone and Joint Research Centre, Chang Gung Memorial Hospital, Taoyuan City 333423, Taiwan; 7 Department of Mechanical Engineering, Ming Chi University of Technology, New Taipei City 24301, Taiwan

**Keywords:** biodegradable polymers, sustainable materials, poly(L-lactide-co-D, L-lactide), β-tricalcium phosphate, electrospinning, barrier membrane, and bone regeneration

## Abstract

In regenerative periodontal treatment, barrier membranes restore periodontal support and aid tissue healing, but slow hard tissue regeneration can disrupt healing and cause tooth instability. This study aimed to fabricate a periodontal membrane through electrospinning poly(L-lactide-co-D, L-lactide) with varying β-tricalcium phosphate (β-TCP) percentages (0%, 10%, 30%, and 40%) treated with hyaluronic acid to enhance bone regeneration in alveolar bone defects. Their ability to promote biomimetic mineralization was characterized using field emission scanning electron microscopy (FESEM) analysis, wettability, and mechanical properties. Biocompatibility and osteogenic differentiation were evaluated by examining BMSCs’ behavior. In vivo, the PLA/β-TCP membrane’s potential to promote bone regeneration was assessed through CT imaging and histological examination. FESEM analysis revealed β-TCP agglomerations within PLA fibers, increasing tensile strength. Water contact angle measurements showed better wettability and higher cell viability after hyaluronic acid treatment, indicating non-cytotoxicity. Membranes with 10% and 30% (*w*/*w*) β-TCP significantly enhanced cellular activities, including proliferation and osteogenic differentiation. Animal tests showed a significant bone growth rate increase to 28.9% in the experimental group compared to 24.9% with the commercial product Epi-Guide after three months. Overall, PLA with 30% β-TCP optimally promoted periodontal hard tissue repair and potentially enhanced bone regeneration.

## 1. Introduction

The oral cavity’s structure includes periodontal tissues essential for maintaining tooth stability and support [1]. The periodontium includes both soft (gingiva, periodontal ligaments) and hard (alveolar bone, cementum) tissues that secure tooth attachment to bones and protect blood vessels, nerves, and teeth from damage [2,3]. These soft and hard tissues are significant for the efficacy of periodontal treatments [4]. The goal of periodontal treatment is the complete healing of both soft and hard tissues in periodontal defects while minimizing the number of surgical procedures. To have a successful reconstruction of the periodontal, cells that have the ability to form cementum, periodontal ligaments, and bone should be able to move to the defect site and activate differentiation to help regenerate bone [3]. Soft tissues around bone defects can invade due to their rapid growth, hindering the cementum’s ability to connect collagen fibers supporting tooth placement. Guided bone regeneration (GBR) membranes effectively isolate bone defects, enhancing bone regeneration [5,6,7].

Guided bone regeneration (GBR) is a surgical technique that stimulates the growth of new alveolar bone in edentulous regions [8]. It involves using techniques of bone grafting and ridge augmentation at the periodontal defect. With the advancement of technology, the periodontal barrier membrane can be applied as guided tissue regeneration (GTR) and bone regeneration (GBR). An ideal barrier membrane should be easy to use, promote tissue adhesion, block soft tissue invasion, and exhibit biocompatibility [9]. One key trait of a GBR membrane is its ability to mimic the natural extracellular matrix (ECM) and enhance osteogenic differentiation [10]. Among the various fabricating techniques for barrier membranes, electrospinning has garnered attention in tissue engineering and dental applications [11]. This technique can create nanofibrous structures that mimic the natural ECM. The versatility of electrospinning techniques allows the use of a wide variety of polymers for various applications, most importantly in biomedical fields [12].

Fabricated barrier membranes using electrospinning technique can be classified as non-resorbable or resorbable. Non-resorbable membranes offer stability but require invasive removal later in healing [13,14,15]. To address this constraint, resorbable membranes, including potentially biodegradable synthetic polymers based on aliphatic polyesters, such as poly(ε-caprolactone) (PCL) [16], poly(glycolic acid) (PGA) [17], poly(lactic acid) [17,18], or their copolymers, that match the resorption time in clinical applications are being utilized. Among these copolymers, poly(L-lactide-co-D, L-lactide), which is denoted as PLA, is flexible and has an improved rate of degradation and has been successfully used as nanofibrous scaffolding for ex vivo cultivation [18] and bone fixation implants [19]. Despite PLA’s remarkable characteristics, its mechanical properties, wettability, and cell attachment remain significant challenges for improvement.

Recently, membranes made of copolymers with ceramic nano additives have led to the development of a new generation of materials for guided tissue regeneration (GTR) and guided bone regeneration (GBR). Ceramic nano additives such as hydroxyapatite and β-tricalcium phosphate (β-TCP) have been identified as biocompatible [20], and they enhance bone growth due to their osteoconductive and highly absorbable characteristics. By adding hydroxyapatite to the copolymer, the electrospun PLA with HA has improved mechanical properties and density values, similar to natural cortical bone [19]. Meanwhile, β-TCP is one of the most widely used and adequate synthetic bone graft substitutes [21].

In a previous study, the electrospun copolymer poly(lactic-co-glycolic acid) was investigated, revealing that the addition of β-TCP led to increased cell proliferation and adhesion [22]. Tammaro et al. [23] explored the potential application of poly(L-lactide-co-D, L-lactide) and β-TCP as a non-woven mesh for hard tissue repair. Alkaline phosphate (ALP) activity enhancement occurred in the non-woven mesh using 10% *w*/*w* β-TCP and decreased when 20% *w*/*w* β-TCP was added after 14 days of culture. β-TCP has been recognized as an outstanding bioceramic widely used in bone reconstruction and dental membrane applications. Thus, it was hypothesized that integrating β-TCP into copolymer PLA membranes could yield significant mechanical stability for preserving the space required for bone regeneration. Additionally, the osteoconductive characteristic of β-TCP might accelerate the proliferation of cells in the periodontal hard tissue, thereby enhancing the efficiency and speed of bone defect healing.

In this study, we fabricated a barrier membrane by preparing different blends of poly (L-lactide-co-D, L-lactide) (PLA) and β-TCP at various concentrations (0%, 10%, 30%, and 40% by weight relative to PLA). These blends underwent an electrospinning process, followed by post-treatment with a 1% hyaluronic acid solution. The study thoroughly examines the electrospun membranes’ morphology, biocompatibility, hydrophilicity, and mechanical properties that could promote bone and tissue regeneration. The potential use of the electrospun membrane in guided bone regeneration was evaluated by assessing osteogenic differentiation. Lastly, a clinical trial comparing the developed electrospun membrane to the commercial product Epi-Guide^®^ (Curasan, Inc., Wake Forest, NC, USA) was conducted in a rabbit’s alveolar bone defect.

## 2. Materials and Methods

### 2.1. Materials

A commercial Poly-L-lactide-Poly-D, L-70:30 Resomer LR 704S (Mw 331,000 g/mol) and PLA + β-TCP 70:30 Resomer LR706 S β-TCP composite were procured from Evonik Industries, Germany. Tricalcium phosphate hydrate (β-TCP) with a particle size of less than 200 nm was obtained from Sigma-Aldrich, Inc. (St. Louis, MO, USA). Methyl ethyl ketone (MEK) of ACS grade was purchased from EMSURE^®^ (Darmstadt, Germany). Hyaluronic acid (HA) with a molecular weight (Mn) of 740,000 g/mol was sourced from Kewpie Corporation (Tokyo, Japan).

### 2.2. Sample Preparation

A copolymer PLA with a concentration of 10% (*w*/*w*) was dissolved in MEK. Composite solutions were then prepared with different concentrations of β-TCP, specifically 0%, 10%, 30%, and 40% (*w*/*w*). For the 40% (*w*/*w*) β-TCP solution, a higher concentration was achieved by adding β-TCP nanopowder (<200 nm) to the composite solution. The solution was prepared at room temperature (25 °C) and magnetically stirred for one day.

The composite solution was loaded into a 20 mL plastic syringe (Luer lock syringe, TERUMO Taiwan Medical Co., Ltd., Taipei City, Taiwan). This solution was fed at a constant rate of 8 mL/h. A pump system (NE-300, New Era) with a Teflon tube connecting the syringe and an 18G needle set up vertically was used for the electrospinning procedure. The synthesis was carried out using a high-voltage power supply to generate an electric field of 14 kV between the collector and the spinneret. Under high voltage, the polymer fibers were generated from the tip and traveled to the grounded collector, forming a micro/nanofiber mesh. The electrospun membrane was collected on a rectangular iron plate covered with baking paper, located 16 cm from the needle tip for optimal membrane deposition.

### 2.3. Electrospun Membrane Post-Treatment

During the initial phase of the experiment, the electrospun membrane showed a persistent hydrophobic surface. According to Abatengelo et al. [24], the aqueous solution of hyaluronic acid (HA) has demonstrated potential in enhancing hydration attributes and has recently been proposed to increase hydrophilicity. The solution was prepared by dissolving 1 g of HA in a solvent composed of 50 g of RO water and 50 g of 95% alcohol. The electrospun membrane was fully immersed in the HA solution to ensure comprehensive absorption of HA. Following this, the membrane was subjected to air drying for 24 h to allow for the evaporation of residual moisture.

### 2.4. Characterization of the Membrane

The electrospun membranes underwent microscopic observation and compositional analysis using a field emission scanning electron microscope (FE-SEM, JEOL JSM-7610FPlus, JEOL Ltd., Tokyo, Japan) at a magnification of ×1000. The analysis assessed the morphology, size of the fiber, presence of defects, and ceramic nanoparticle adhesion to the fibers.

### 2.5. Water Contact Angle Measurement

Samples were evaluated using an experimental setup specifically developed and constructed in our laboratory to measure the water contact angle and verify the hydrophilicity of the electrospun membrane. The electrospun membrane sample was positioned on the device’s platform, with the focus adjusted to the membrane’s surface. A water droplet of approximately 2 μL was then dispensed onto the surface of the membrane. After a duration of 30 s, the contact angles were calculated by drawing tangents on the water-substrate interface and water-air interface, measured with the device. The state of the water droplet on the surface was subsequently captured using the device’s photographic capabilities.

### 2.6. Adhesion, Water Absorption, and Filament Shedding Properties

The adhesion test was initiated by cutting the membrane immersed in a hyaluronic solution, as detailed in Section 2.3. The membrane was then cut into 1 cm × 1 cm pieces and fully saturated with water. The saturated membrane was then affixed to the second joint of the index finger to assess its adhesion and record the membrane texture. The water absorption test involved immersing the membrane in water, with tweezers aiding in water absorption and air bubble removal. The time the membrane took to absorb water and its post-absorption state were recorded fully. Lastly, the fiber shedding test was conducted by gently rubbing the entire membrane after the water absorption test to check for detaching fibers. These tests collectively offer valuable insights into the properties of the hyaluronic acid-soaked membrane.

### 2.7. Mechanical Properties

The mechanical properties of the electrospun membrane were evaluated using a universal testing machine. The membrane was carefully cut into the desired shape (like a bone) with specific dimensions. The sample was then inspected for defects and bursts in the necking area, which could potentially influence the mechanical strength data. Ends were marks at 1 cm intervals on both sides (Figure S1), and the thickness of the necking area, the width of the necking area, and the distance between the two marks (gauge length) were documented. The sample was subjected to equal but opposite forces at 12.5 mm/min and 1 mm/min speeds until completely ruptured. For data analysis, the tensile stress (σ) and breaking strain were calculated using the following equations [25]:

σ = (Max Load (N))/(Neck thickness (mm) × Neck width (mm)),(1)

strain (%) = gauge length (mm)/deformation (mm)(2)

### 2.8. Cytotoxicity Test

The cytotoxicity test (indirect method) of the membrane sample was evaluated using the CCK-8 Kit (UNI-ONWARD Corp.) assay with a leaching pattern in accordance with the manufacturer’s instructions. Three membrane samples measuring 1 cm × 1 cm each were placed on a 24-well plate, according to the diagram in Figure S2. The culture medium (500 λ) was added to each well containing a membrane sample and two empty wells. The 24-well plate was placed in a cell culture incubator at 37 °C with 5% CO_2_ for three days. Afterward, the extraction solution was retrieved from each well.

Rat bone marrow stromal cells (BMSCs) were then seeded in a 96-well plate at a density of 2 × 10^3^ cells per 100 μL with four replicates (see Figure S3). The plate was incubated overnight at 37 °C with 5% CO_2_ in a cell culture incubator to ensure cell adhesion. The culture medium was then removed from the 96-well plate, and the extraction solution from the 24-well plate was added to each well, with a volume of 100 μL per well, according to their respective parameters. The plate was returned to the incubator at 37 °C with 5% CO_2_ for three days. Following this, 1% Triton-X-100 was added to the plate count (PC) wells, and the plate was incubated for an additional 10 min. The medium in the 96-well plate was then removed, and each well received 100 μL of a prepared 10% CCK-8 solution, as per the diagram (Figure S4). The plate was incubated for one hour at 37 °C with 5% CO_2_, after which the absorbance was measured at a wavelength of 450 nm.

### 2.9. Osteogenic Differentiation Test

The osteogenic differentiation was evaluated via quantitative alkaline phosphatase (ALP) activity assay. The experimental setup was in accordance with the laboratory protocol and the osteogenic induction medium manufacturer’s instructions. The direct cell osteogenic differentiation test procedure involved placing two 1 cm × 1 cm membrane samples per parameter into a 24-well plate. Rat bone marrow stromal cells (BMSCs) were then seeded onto each membrane at a density of 3 × 10^4^ cells per 60 μL, as shown in Figure S5. The plate was then incubated in a cell culture incubator at 37 °C with 5% CO_2_ for one hour to facilitate cell adhesion to the membrane.

Upon confirmation of cell adhesion, an additional 500 μL of culture medium was added, and the plate was incubated overnight at 37 °C with 5% CO_2_. The culture medium in the 24-well plate (Figure S6) was then replaced with an osteogenic induction medium. The plate was returned to the cell culture incubator at 37 °C with 5% CO_2_, and the medium was changed every 2–3 days over a total culture period of 14 days. After 14 days, the culture medium in the 24-well plate was discarded, and the cells were rinsed with phosphate-buffered saline (PBS). The cells were then fixed with formalin, rinsed again with PBS, and a 5 mg/mL pNPP (p-nitrophenyl phosphate) (SIGMA) solution was prepared and added to the membrane samples at a volume of 500 μL. The plate was kept in a dark area at room temperature for 45 min. Subsequently, absorbance values were measured at a wavelength of 405 nm using a spectrophotometer, with 100 μL of each sample transferred to the absorbance measurement plate, according to their respective parameters.

### 2.10. In Vivo Study

A clinical simulation was carried out using sixteen rabbits as the subject of the experiment. The animal use protocol was reviewed and approved by the Institutional Animal Care and Use Committee (IACUC), and the Committee recognized that the proposed animal experiments followed the *Animal Protection Law* of the Council of Agriculture. The established surgical protocol involved creating a circular bone defect in the rabbit’s mandibular alveolar bone. This defect was precisely 1 cm in diameter. The animals were then allocated into three independent groups: (i) a blank control group (no treatment), in which the bone defect was created and the rabbits were immediately sutured; (ii) a control group, in which a commercial Epi-Guide was used; (iii) an experimental group, in which the developed periodontal regeneration membrane was used.

The developed membrane was initially cut into circles approximately 1.5 cm in diameterand inserted into the bone defect. This was followed by the addition of roughly 0.5 cm^3^ of bone graft material. Afterward, another piece of the periodontal regeneration membrane was placed atop the bone defect, and a bone plate was positioned beneath the bone defect to prevent bone deformation or fracture. Finally, the surgical site was sutured. Three months after the operation, the rabbit’s mandibular alveolar bone was evaluated for its osteogenic effect. A high-resolution CT scan was performed to evaluate new bone formation within the mandibular alveolar bone. Sections stained with Aniline blue and embedded in methyl methacrylate were performed following the manufacturer’s instructions.

## 3. Results and Discussion

**Figure 1:**
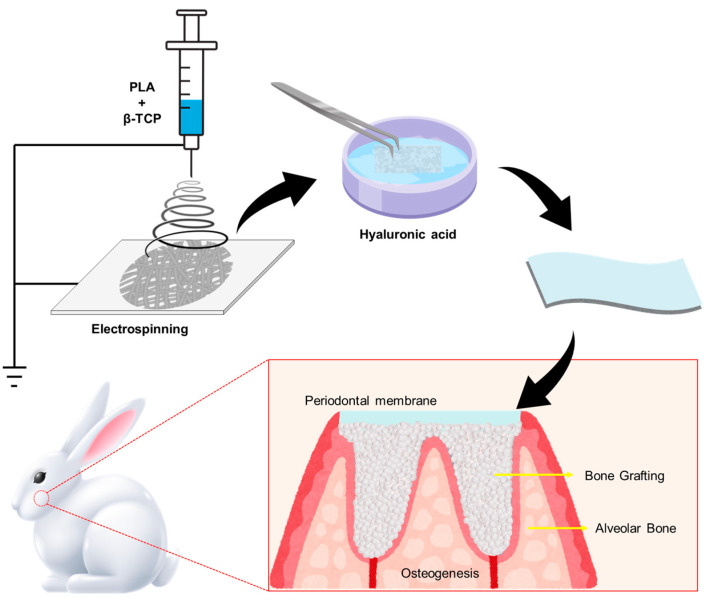
Fabrication and working mechanism of the electrospun PLA/β-TCP periodontal membrane for alveolar bone regeneration.

The framework and working principle of the barrier membrane, as shown in Figure 1, involved preparing a blended PLA/β-TCP membrane using electrospinning technology and soaking it in a hyaluronic acid solution. In vitro experiments confirmed that the developed membrane is a non-toxic material and can accelerate bone cell growth. Subsequently, the membrane was implanted in a rabbit’s alveolar bone defect model to assess bone regeneration.

### 3.1. Surface Morphology/Composition Analysis

**Figure 2:**
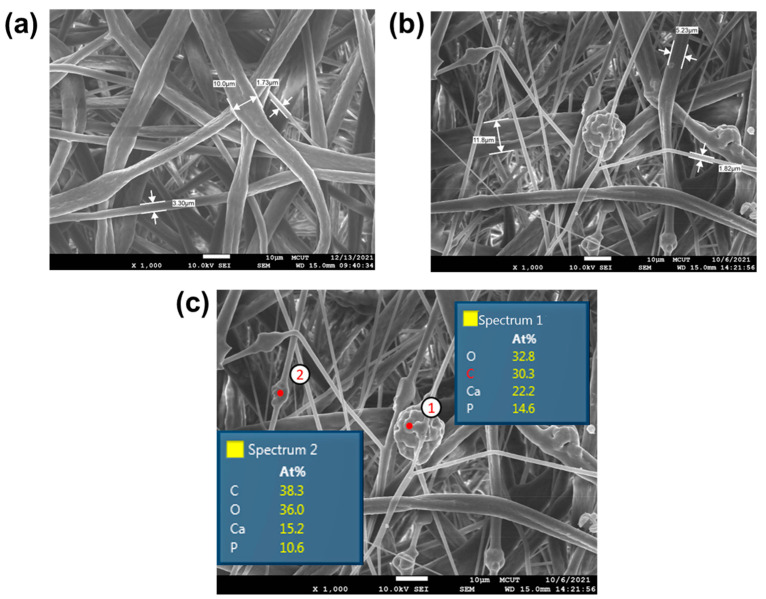
Electrospun PLA membrane characteristics. (**a**) Morphology of pure PLA and (**b**) PLA + 30% β-TCP. (**c**) Elemental mapping of PLA + 30% β-TCP, as shown by FESEM at a magnification of ×1000.

The surface morphology of the PLA membranes was analyzed using FESEM. The results indicated that both pure PLA and PLA with a percentage of β-TCP powder exhibited non-uniform fiber formation. Pure PLA could form fibers within a range of 1.7–10 μm (Figure 2a), while the addition of 30% β-TCP resulted in fiber diameters of 1.8–12 μm (Figure 2b). The surface morphology and diameters of the produced nanofibers were assessed using FESEM analysis. The results indicated that both pure PLA and PLA with β-TCP powder exhibited randomly oriented, non-uniform fiber formation. Due to the high volatility of MEK, using it as a single solvent for electrospinning often led to nozzle blockages, hindering the smooth progress of the spinning process. The emission of charged jets was intermittent, a direct result of the drying out of the pending droplets at the spinneret tip because of the low boiling point of MEK [26]. This resulted in large diameters and poor uniformity of the fiber diameter.

The effect of highly volatile solvents was also observed in Yang’s study [27], where poly(L-lactic acid) solutions mixed with the highly volatile solvent DCM (Dichloromethane) experienced non-uniform fiber diameter. The authors reported that using a highly volatile solvent caused the solution to dry quickly at the spinneret tip, potentially leading to defects and a non-uniform size of the fiber.

It was also observed that membranes with β-TCP led to agglomeration in the fiber [28,29]. Some β-TCP is encapsulated with the fiber (point 2), and some are exposed (point 1). The EDS-point analysis detected calcium and phosphorus components at both points, and the ratio of calcium to phosphorus was 3:2, suggesting the presence of β-TCP. Point 1 showed higher calcium and phosphorus content than point 2, which can be attributed to the encapsulation of β-TCP particles within PLA fibers at point 2. As PLA fibers primarily comprise carbon and oxygen, point 2 displayed higher carbon and oxygen content than point 1.

### 3.2. Water Contact Angle of the Membrane

The surface water contact angle measured by the drop method was used to evaluate the hydrophilicity of the samples. The electrospun membrane initially displayed a hydrophobic surface, indicating the need to soak it in a hyaluronic solution. As presented in Table 1, the membrane exhibited an average weight increase of approximately 0.018 g after being soaked and air-dried. This 25.4% weight increase indicated that HA material adhered to the membrane following post-treatment (Table S1).

**Figure 3:**
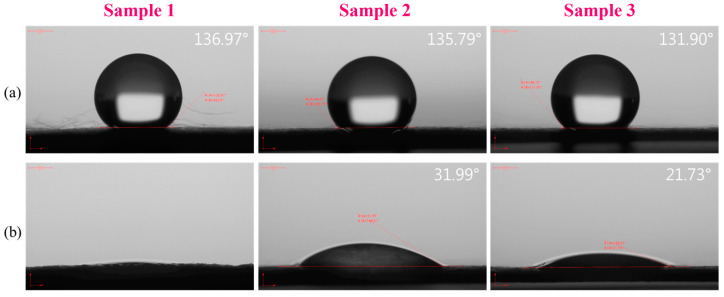
Water contact angle result of PLA + 30% β-TCP membrane samples. (**a**) The state prior to immersion and (**b**) after immersion in a hydrophilic additive solution.

Water contact angle (WCA) measurements were used to evaluate the wettability of all sample membranes. All sample membrane surfaces had contact angles exceeding 120 degrees, signifying a superhydrophobic state [30], as shown in Figure 3a. However, the contact angle of the electrospun membrane samples significantly decreased to <10°, 31.99°, and 21.73° respectively, after immersion in a hydrophilic additive, indicating a shift to a completely wetting, hydrophilic surface. Previous studies have shown that HA possesses adhesive properties and is well-known for its hydrophilic nature, which allows it to retain water molecules [31,32]. When the substrate was dipped in an HA solution, it significantly enhanced its surface roughness. This surface modification also reduced the contact angle, indicating improved wettability and hydrophilicity.

### 3.3. Adhesion, Water Absorption, and Filament Shedding Properties

**Figure 4:**
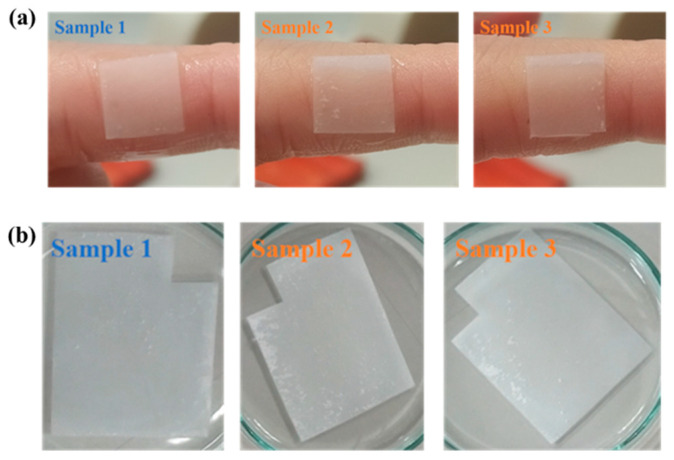
(**a**) Results of the adhesion test for PLA + 30% β-TCP electrospun membranes and (**b**) water absorption test for electrospun membranes. The time required for sample 1, sample 2, and sample 3 membranes to be saturated is 23.29 s, 28.53 s, and 27.23 s, respectively.

A barrier membrane should possess excellent adhesion properties to ensure compatibility and adherence with periodontal tissue. In clinical applications, the membrane is hydrated with a solution to enhance its adhesiveness before placing it at the affected site. Therefore, this study used water as a hydrating solution to test the adhesiveness of the electrospun membrane. As observed in Figure 4a, Sample 1 can fully adhere to the fingertip, while the upper parts of Sample 2 and Sample 3 do not entirely adhere. This difference is attributed to the texture; Sample 1 has a loose and soft texture, while Sample 2 and Sample 3 have a firmer texture. The weight data from Table 2 of the electrospun membrane further supports this observation. Sample 1, with its loose and soft texture, exhibits the highest weight difference (0.0082 g), whereas the firmer membranes show weight differences of only 0.0056 g and 0.0055 g. These results suggest that softer membranes retain more hyaluronic acid material, leading to better adhesion. Although all the samples followed the same parameters, the environmental conditions varied due to the uncontrolled environment of the electrospinning chamber. The deviation in texture may have been caused by inconsistent humidity and temperature, which affect how the jet is deposited on the collector. According to Mailley et al. [33], humidity plays an important role in the evaporation rate of solvent. Residual solvents allow fiber–fiber adhesion, and due to the rapid solidification of droplets, the amounts of residual solvents on the fiber surface are reduced. This reduction causes fewer fibers to bind together, resulting in a fluffy, cotton-like mesh structure.

The water absorption capabilities of electrospun membranes are critical for their efficacy as a periodontal membrane. These properties facilitate the easy diffusion of nutrients and metabolites between cells and membranes [34]. The water absorption rates of the electrospun membranes were evaluated and timed, as shown in Figure 4b. The times required for the membranes to be fully saturated were determined to be 23.29 s, 28.53 s, and 27.23 s for Sample 1 (loose and soft texture), Sample 2 (firm texture), and Sample 3 (firm texture) membranes, respectively. It can be observed that a loose and soft-texture membrane requires less time to absorb water than a firm-texture membrane. This is because, after soaking it in a hyaluronic acid (HA) solution, Sample 1 gained the most weight, as shown in Table 2. The increased weight indicates that more HA material was attached to it, resulting in faster water absorption and more complete saturation.

According to the results, membranes with loose and softer textures exhibit better adhesion. After soaking, these membranes retain a higher residual hyaluronic acid content, improving water absorption. It is important to monitor the membrane for excessive looseness during testing, as this may lead to filament shedding. However, gentle rubbing revealed no significant fiber shedding in any of the membranes after the water absorption test. On the contrary, adhesion was poorer when the membrane texture was more rigid, and the residual hyaluronic acid content on the membrane after soaking was lower, resulting in poorer water absorption.

### 3.4. Mechanical Properties of the Membrane

**Figure 5:**
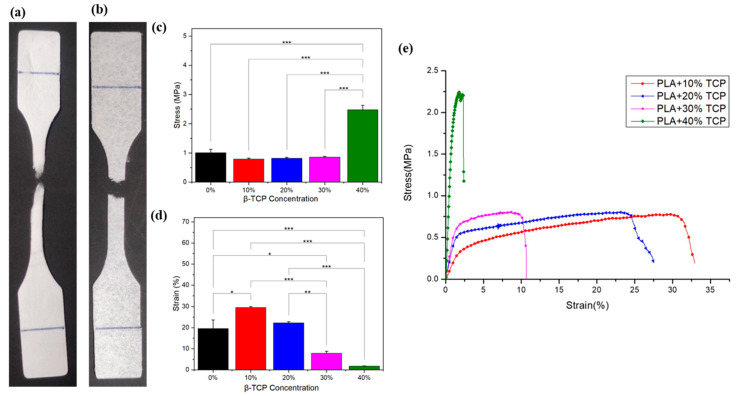
Mechanical properties of electrospun membranes. (**a**) Membranes with lower calcium phosphate (β-TCP) content exhibit ductility, (**b**) With a higher calcium phosphate (β-TCP) content, the membrane becomes brittle. (**c**) The tensile strength and (**d**) breaking strain of membranes with different ratios of calcium phosphate (β-TCP) powder were measured at a stretching speed of 12.5 mm/min (*n* = 3). (**e**) Representative stress–strain curves of electrospun membranes. Data were analyzed using *t*-tests. * *p* < 0.05, ** *p* < 0.01, *** *p* < 0.001, statistically significant difference.

Dentists typically fill bone defects with bone graft material during periodontal surgery to maintain space. Subsequently, they overlay and suture a periodontal regeneration membrane onto the defect. Therefore, the membrane needs to have a certain mechanical strength to prevent damage during the surgical procedure. As shown in Figure 5c, pure PLA demonstrates a higher tensile strength, averaging 1.014 MPa, than PLA with 10%, 20%, and 30% concentrations of β-TCP. As the β-TCP concentration increases, the tensile strength also improves: 0.818 MPa for 20% β-TCP and 0.859 MPa for 30% β-TCP (Table S2). Notably, when 40% β-TCP is added, the tensile strength increases significantly. The produced membrane demonstrated the highest tensile strength, averaging 2.477 MPa across three trials, and the breaking strain was 1.818%, as shown in Figure 5d. Also, the stress–strain curve is shown in Figure 5e. The results indicate an inversely proportional relationship between tensile strength and elongation at break in the electrospun membranes. As the tensile strength of the membrane increases, its resistance to deformation also increases, resulting in a reduced ability to stretch significantly before breaking. Conversely, membranes with lower tensile strength exhibit higher elongation at break, demonstrating greater stretchability before failure.

Similar results were demonstrated in other research, which demonstrated that the mechanical properties improved after adding tricalcium phosphate nano powder [35,36]. Additionally, mechanical testing revealed that membranes with lower concentrations of β-TCP exhibit ductile behavior, as shown in Figure 5a. However, as the concentration increases, the membrane’s characteristics shift toward brittleness, as depicted in Figure 5b. This behavior may arise from β-TCP agglomeration within the fibers, hindering the connection between PLA fibers. Consequently, the transition from ductility to brittleness leads to a significant decrease in strain values over time, as shown in Figure 5d.

Under consistent electrospinning processing and solution parameters, slight environmental variations can alter the membrane’s texture, thereby affecting its mechanical strength characteristics. Specifically, slower solvent evaporation during spinning in environments with higher humidity leads to enhanced bonding between fibers, resulting in a firmer membrane texture and increased mechanical strength. In Table 3, the summary of the mechanical strength results reveals that electrospun membranes with a loose and soft texture exhibit an average tensile strength of 0.780 MPa and a strain value of 11.538%. This weaker bonding between fibers makes them more susceptible to deformation and breakage. Conversely, when the membrane texture is firm, stress–strain values increase, with an average range of 1.118–1.136 MPa and a breaking strain of 22.5–24.7%. Notably, the correlation between fiber bonding and mechanical strength underscores the importance of environmental parameters in electrospun membrane development. Higher humidity environments favor stronger bonding during spinning, enhancing the membrane’s mechanical properties. The experimental data is in Supplementary Table S3.

### 3.5. The Impact of Hydrophilicity/Hydrophobicity on Cell Experiments

**Figure 6:**
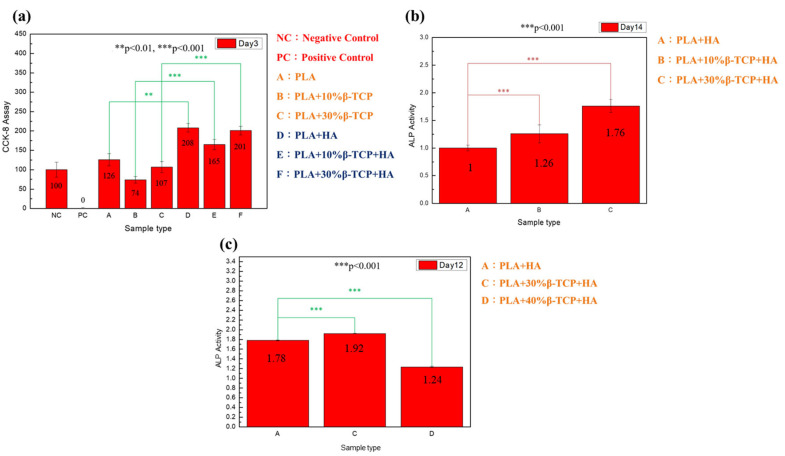
Cellular behavior of BMSCs cultured on electrospun membrane: (**a**) viability of BMSCs in 500λ culture medium on day 3, (**b**) ALP activity during the first trial of the osteogenic differentiation test on day 14, (**c**) ALP activity during the second trial of the osteogenic differentiation test on day 12. Data were analyzed using two-way *t*-tests. ** *p* < 0.01, *** *p* < 0.001, statistically significant difference.

One of the most important criteria for a tissue engineering membrane is its hydrophilicity. Water uptake capacity and hydrophilicity properties directly affect cell adhesion and hydrolytic degradation [37]. The toxicity of the medium, which contains substances released from electrospun membranes with different parameters, was evaluated by calculating scores for each sample. The scores of the cytotoxicity test showed that the addition of β-TCP to pure PLA results in a decrease in cell viability (Figure 6a). Despite this, the results indicate that it is not critically cytotoxic, as all samples maintained over 70% viability [38].

Liu et al.’s [39] study suggests a potential for slight toxicity, with β-TCP slightly reducing cell viability depending on the concentration. However, decreasing cell viability to as low as 70% does not imply that β-TCP utilization is highly toxic. This suggests that the substances released from the electrospun membranes are non-toxic to the cells. In addition, an increase in cell viability was observed by decreasing the percentage of β-TCP and soaking the membranes in HA. The HA enhanced the hydrophilicity of the membrane, which increased the adhesion tendency of fibroblast cells and led to greater cell proliferation. Maintaining high viability and active proliferation of cells is essential to promote successful osteogenesis in bone regeneration.

The electrospun membranes immersed in HA showed a significant increase in cell viability [40]. The HA in an aqueous solution consists of negatively charged molecules due to the presence of the carboxylic group. This group is recognized for its pronounced hydrophilicity under physiological pH conditions. As a result, the electrospun membrane immersed with HA has a significant capacity to attract and bind a large number of water molecules. These molecules form hydrogen bonds with the carboxylic group of HA through intramolecular and intermolecular bonding [41,42]. Furthermore, a *t*-test statistical analysis indicated a significant increase in cell viability for samples immersed in hyaluronic acid compared with those not immersed, with *p*-values ranging between 0.008 and 0.0005 (* *p* < 0.05, ** *p* < 0.01, *** *p* < 0.001), demonstrating a significant enhancement in biocompatibility.

### 3.6. The Impact of Tricalcium Phosphate (β-TCP) Powder Addition on Cell Experiments

The osteogenic differentiation resulting from the membrane and osteogenic medium was investigated and evaluated using alkaline phosphatase (ALP) activity. ALP is a well-known early marker for osteogenic differentiation and can be quantified using the absorbance values measured from the samples. After obtaining absorbance values, the absorbance of control group A (PLA + HA) was set to 1. The scores for samples B (PLA + 10% β-TCP + HA) and C (PLA + 30% β-TCP + HA) from the initial osteogenic differentiation test were calculated and are depicted graphically in Figure 6b, with the data recorded on day 14 of culture. A second osteogenic differentiation test was conducted, introducing an additional sample D (PLA + 40% β-TCP +HA), as illustrated in Figure 6c. The ALP activity in the PLA + HA membrane, when supplemented with β-TCP nanopowder, corresponded to an increase in cell proliferation compared with the pure PLA + HA. As the proportion of β-TCP powder increased (as shown in Figure 6b,c), the bone differentiation also increased. Statistical analysis using a *t*-test comparing samples with and without β-TCP powder addition showed a significant increase, with *p*-values less than 3.4 × 10^−5^ (*** *p* < 0.001), indicating a significant enhancement in bone cell growth. Specifically, PLA + 30% β-TCP/HA exhibited significantly higher ALP levels, with increases of 1.76- and 1.92-fold. However, the PLA + 40% β-TCP/HA group showed a significant decrease in ALP activity compared with the lower concentration of β-TCP. Statistical analysis using a *t*-test comparing samples with 40% β-TCP powder addition to those without β-TCP powder addition shows a significant decrease, with a *p*-value of 1.4 × 10^−4^ (*** *p* < 0.001), indicating a significant reduction in osteogenic differentiation.

In this study, the PLA membrane containing 30% β-TCP/HA exhibited a favorable cellular response regarding cell attachment and proliferation. Several factors contribute to the electrospun membrane’s cell activity. Previous studies have confirmed that β-TCP can directly or indirectly enhance osteoblast differentiation, promote vascularization, regulate the release of angiogenic growth factors, and influence blood clot formation through the release of ionic components like Ca^2+^ and PO_4_^3−^ [43,44,45,46]. When β-TCP is introduced into a wound or bone defect, it interacts with blood components, triggering clotting mechanisms and providing natural structural support during hard tissue healing, thus improving the healing process [47]. However, excessive β-TCP may lead to dense blood clots of thin and dense fibrin, which can be highly resistant to fibrinolysis, potentially delaying bone healing [48]. This occurrence suggests that the excessive addition of β-TCP powder results in a rapid decrease in cell osteogenic differentiation, and the optimal amount of β-TCP addition is 30%.

The enzymatic activity of BMSCs in the pure PLA membrane was further improved by blending the material with β-tricalcium phosphate (β-TCP) to create a membrane. Calcium and phosphate groups in β-TCP have been identified as significant groups in promoting osteogenic differentiation. Additionally, the formation of a calcium-phosphorus deposition layer enhances cell adhesion on the fiber surface [49]. This behavior promotes faster cell movement, which helps regenerate the damaged periodontal hard tissue.

### 3.7. Alveolar Bone Regeneration In Vivo

**Figure 7:**
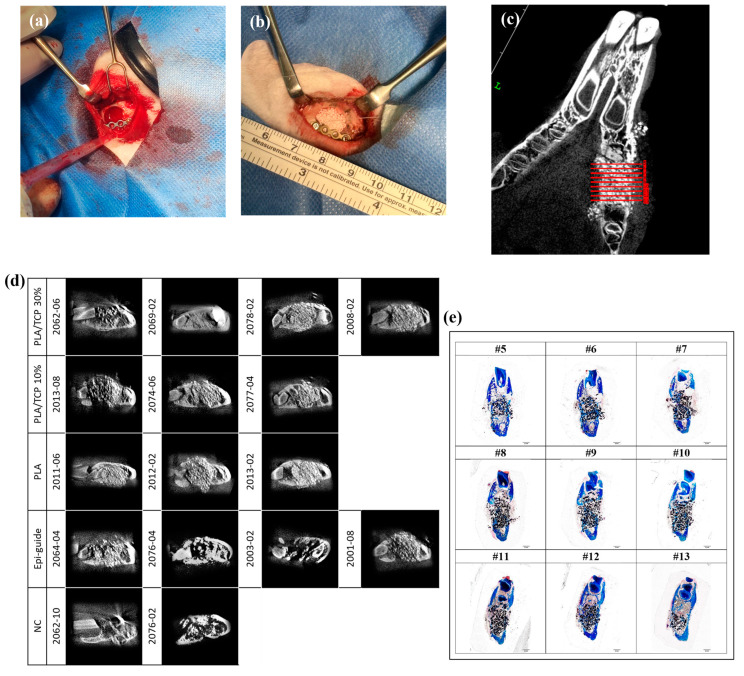

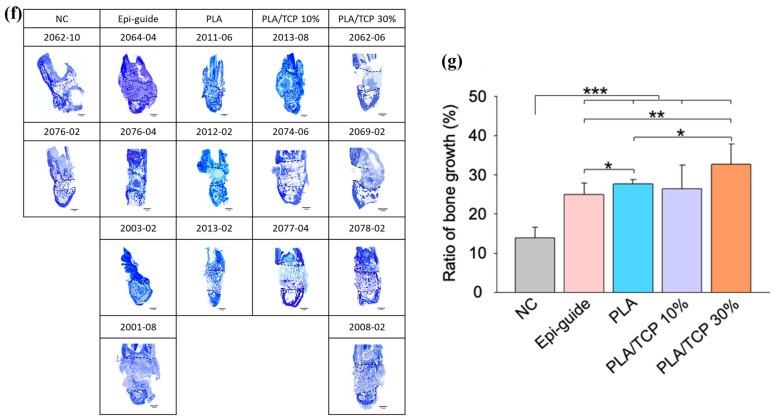
In situ bone regeneration in the rabbit alveolar bone defect model. (**a**) Circular bone defect with a diameter of 1 cm created in the mandibular alveolar bone of rabbits, then (**b**) placement of bone grafting material and membrane. (**c**) Selection of area (3 mm × 3 mm) for bone growth rate calculation. (**d**) CT images of specimens of alveolar bone defect tissues at postoperative month 3 (coronal view). (**e**) Photomicrographs of methyl methacrylate-embedded sections of the alveolar bone defect tissues treated with PLA + 30% β -TCP at postoperative month 3. (**f**) Aniline blue staining of the alveolar bone defect tissues at postoperative month 3. (**g**) Ratio of bone growth of the negative control (NC), commercial Epi-guide, and developed electrospun membrane. Data were collected using *t*-test. * *p* < 0.05, ** *p* < 0.01, *** *p* < 0.001, statistically significant difference.

An animal experiment was conducted to confirm the clinical effectiveness of the developed periodontal regeneration membrane. A circular bone defect in rabbit mandibles was successfully created (Figure 7a) and subsequently treated using a membrane and bone graft (Figure 7b). In addition to the negative control (NC) group, the animal experiment included samples prepared from the commercial product Epi-Guide. The test on the rabbit was reported through CT imaging, as shown in Figure 7d, and histological examination of alveolar bone defects stained with Aniline blue (taken three months post-operation). These images were analyzed to provide insights into new bone formation at the damaged periodontal hard tissue site. The data gathered quantitatively assessed the extent of new bone growth within the defect sites. The blank control group exhibited minimal new bone formation 3 months after the operation. By contrast, the experimental groups displayed varying degrees of bone growth, as depicted in Figure 7g. Notably, the PLA+ 30% β-TCP groups showed significantly higher levels of new bone formation compared with both the blank and Epi-Guide groups, indicating a superior osteogenic effect.

The methyl methacrylate-embedded sections revealed consistent findings: significant bone regeneration occurred at 3 months post-operation with PLA + 30% β-TCP treatment. The bone growth area, measured as 3 mm × 3 mm using Image J software 3.1.52V (Figure 7c), exhibited an average growth rate of 28.9%. Notably, this rate surpassed the 24.9% bone growth observed with the commercially available Epi-Guide treatment (Figure 7g). These results underscore the potential of the PLA + 30% β-TCP periodontal regeneration membrane in enhancing bone growth within alveolar bone defects in the rabbit animal model.

## 4. Conclusions

This study successfully fabricated an electrospun membrane using a blend of poly (L-lactide-co-D, L-lactide) (PLA) and beta-tricalcium phosphate (β-TCP) to optimize material composition for the regeneration of periodontal hard tissue. Morphological analysis revealed that β-TCP appeared as agglomerations within the smooth fibers, which had diameters ranging from 1.8 to 12 μm, resulting in defect-free fibers. The study found that increasing the concentration of β-TCP enhanced the mechanical properties of the membrane. However, a 40% concentration of β-TCP negatively impacted cellular activities, leading to reduced osteogenic differentiation. The optimal combination was a 30% concentration of β-TCP, which balanced mechanical properties and bioactivity. Additionally, the firm texture of the membrane increased its tensile strength compared with the loose fiber-fiber bonding. Soaking the membrane in hyaluronic acid significantly improved its hydrophilicity, leading to better cellular activities, adhesion, and proliferation.

Overall, the incorporation of a 30% concentration of β-TCP improved the membrane’s bioactivity and mechanical properties, with a bone growth rate increase to 28.9% compared with the commercial Epi-Guide’s 24.9%. However, the study has several limitations, including uncontrolled environmental conditions, and further study is needed to optimize the balance between mechanical properties and cellular activity. Nevertheless, the PLA/β-TCP membrane shows promising potential as a barrier membrane for periodontal treatment.

## Figures and Tables

**Table 1:** The weight difference of PLA + 30% β-TCP electrospun membrane before and after soaking in hyaluronic acid for the WCA test; thickness (t), before soaking (BS), after soaking (AS), weight difference (WD).

Sample	t (mm)	BS (g)	AS (g)	WD (g)	WD (%)
1	0.322	0.0707	0.0897	0.0190	26.9
2	0.316	0.0707	0.0879	0.0172	24.3
3	0.321	0.0706	0.0883	0.0177	25.1
Average	0.320	0.0707	0.0887	0.0180	25.4

**Table 2:** The weight difference of PLA + 30% β-TCP electrospun membrane before and after soaking in hyaluronic acid; thickness (t), before soaking (BS), after soaking (AS), weight difference (WD).

Sample	t (mm)	BS (g)	AS (g)	WD (g)	WD (%)	Texture
1	0.237	0.0437	0.0519	0.0082	18.8	Loose and soft
2	0.239	0.0522	0.0578	0.0056	10.7	Firm
3	0.233	0.0500	0.0555	0.0055	11.0	Firm

**Table 3:** Mechanical strength of pure PLA electrospun membrane samples with varying textures at a stretching speed of 1 mm/min. (*n* = 3).

Samples	Force (N)	Thickness (mm)	Width (mm)	Stress (MPa)	Strain %	Texture
1	0.758357	0.303	3.18	0.780	11.538	Loose and soft
2	0.840041	0.223	3.18	1.136	22.532	Firm
3	0.870360	0.244	3.18	1.118	24.745	Firm

## Data Availability

The original contributions presented in the study are included in the article/supplementary material, further inquiries can be directed to the corresponding author.

## Supplementary Materials

The following supporting information can be downloaded at: https://www.mdpi.com/2073-4360/17/3/256/s1: preparation and optimization of the solution, electrospinning process optimization. Figure S1: Sample membrane for mechanical testing. (**a**) Tensile test sample dimension diagram (**b**), sample membrane ready for tensile test; Figure S2: Diagram of the extraction process for the cytotoxicity test (indirect method); Figure S3: Diagram illustrating the Cell Counting Kit-8 assay procedure for the cytotoxicity test (indirect method); Figure S4: Diagram illustrating the seeding of cells for the cytotoxicity test (indirect method); Figure S5: Diagram illustrating the seeding of cells for the direct cell osteogenic differentiation test; Figure S6: Diagram illustrating the ALP assay procedure for the direct cell osteogenic differentiation test; Table S1. Hydrophilicity test results. Weight change before and after soaking the membrane in hyaluronic acid; Table S2. Mechanical strength results were obtained for electrospun membranes with different ratios of calcium phosphate (β-TCP) powder at a stretching speed of 12.5 mm/min; Table S3. Mechanical strength results were obtained for electrospun membranes with different textures at a stretching speed of 1 mm/min.
