# Supplementary material for: Enhancing Bone Regeneration Using Blended Poly(L-lactide-co-D, L-lactide) and β-Tricalcium Phosphate Nanofibrous Periodontal Biodegradable Membranes
Source: Polymers (Basel). 2025 Jan 21;17(3):256. doi: 10.3390/polym17030256 (PMC12128702; doi:10.3390/polym17030256)
Supplement: Supplementary file 1 [file polymers-17-00256-s001.zip › polymers-3397094-supplementary.pdf]

polymers

MDPI

Supplementary Materials

Enhancing Bone Regeneration Using Blended Poly(L-lactide-co-D, L-lactide) and β-Tricalcium Phosphate Nanofibrous Periodontal Biodegradable Membranes

Princess Joy Naig [1], Zih-Yin Kuo [2], Min-Fan Chung [3], Chih-Hao Chen [4,5], Chi-Yun Wang [1,6] and Kuo-Yung Hung [2,7,*]

[1] Department of Biomedical Engineering and Medical Devices, Ming Chi University of Technology, New Taipei City 24301, Taiwan
[2] Research Center for Intelligent Medical Devices, Ming Chi University of Technology, New Taipei City 24301, Taiwan
[3] NAN YA Plastics Corporation, Plastics 1st Division, New Taipei City 238, Taiwan
[4] Department of Plastic and Reconstructive Surgery, Chang Gung Memorial Hospital at Keelung, Keelung 204, Taiwan
[5] Department of Plastic and Reconstructive Surgery, Chang Gung Memorial Hospital at Linkou, Chang Gung University, College of Medicine, Taoyuan 333, Taiwan
[6] Bone and Joint Research Centre, Chang Gung Memorial Hospital, Taoyuan City 333423, Taiwan
[7] Department of Mechanical Engineering, Ming Chi University of Technology, New Taipei City 24301, Taiwan
* Correspondence: kuoyung@mail.mcut.edu.tw

Preparation and Optimization of the solution

In a controlled room environment, the copolymer PLA was prepared at a concentration of 10% (w/w) dissolved in methyl ethyl ketone (MEK). To explore the potential of β-TCP (beta-tricalcium phosphate) in enhancing bone differentiation, various concentrations of β-TCP were initially prepared at 0%, 10%, 30%, 40%, 50%, and 60%. Given the ceramic biomaterial's excellent bone differentiation properties, our objective was to optimize its incorporation into the copolymer matrix. However, during the preparation for electrospinning, it was observed that β-TCP concentrations of 50% and 60% resulted in significant precipitation when mixed with the MEK. This precipitation hindered the smooth formation of fibers, making it impractical to use these higher concentrations. Consequently, only samples with β-TCP concentrations up to 40% were produced for bone differentiation testing. The results indicated that increasing the β-TCP ratio to 40% did not enhance the bone differentiation effect, suggesting that the optimal concentration for this application lies below this threshold.

Electrospinning Process Optimization

The electrospinning process was carefully optimized to ensure the successful formation of fibers. One of the critical parameters was the applied voltage. Initially, a low voltage of 12kV to 13kV was sufficient to create jetting motion of the solution and initiate the spinning process. However, the flow of the solution eventually became intermittent, and frequent solidification of droplets occurred at the spinneret. This issue disrupted the continuous formation of fibers, indicating that the range of 12-13kV was not optimal. Upon further adjustment, it was found that a voltage of 14kV provided a smooth and continuous jetting with minimal solidified droplets. The attempts to increase the voltage further to improve fiber stretching were unsuccessful. Increasing the voltage beyond 14kV caused the fibers to stretch excessively, preventing the incorporation of β-TCP (beta-

tricalcium phosphate) into the fibers. Therefore, maintaining the voltage at 14kV was essential for achieving the desired fiber characteristics and ensuring the successful integration of β-TCP.

Another crucial parameter was the distance between the spinneret and the collector. This distance was optimized within the range of 16-20 cm to ensure proper stretching and formation of fibers. It was observed that a larger distance of 20 cm made it difficult to collect the whipping fibers. The extended flying path and reduced traction force of the electric field hindered the full stretching of the fibers, resulting in a loose fiber structure. Therefore, a shorter distance within the optimized range was preferred to ensure the proper collection and formation of well-structured fibers. By carefully optimizing these parameters, we aimed to achieve the best possible fiber formation and bone differentiation properties in the copolymer-β-TCP composite.

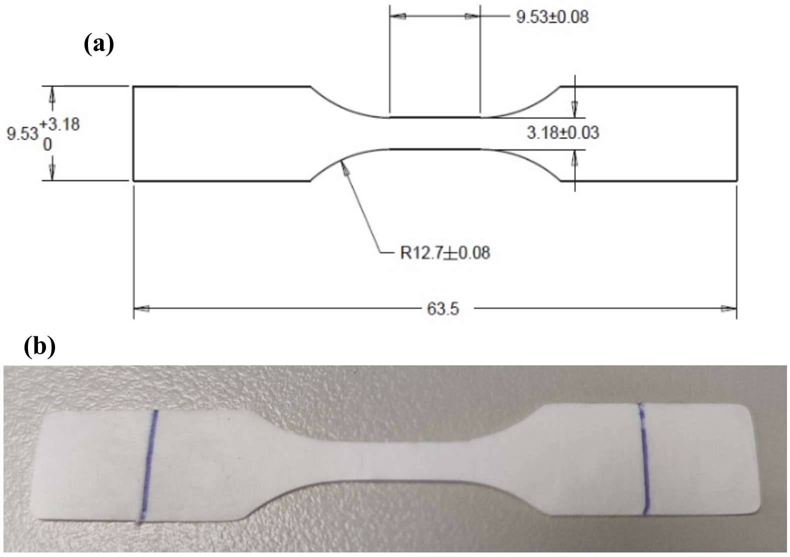

Figure S1. Sample Membrane for Mechanical Testing. **(a)** Tensile Test Sample Dimension Diagram **(b)** Sample Membrane ready for Tensile Test

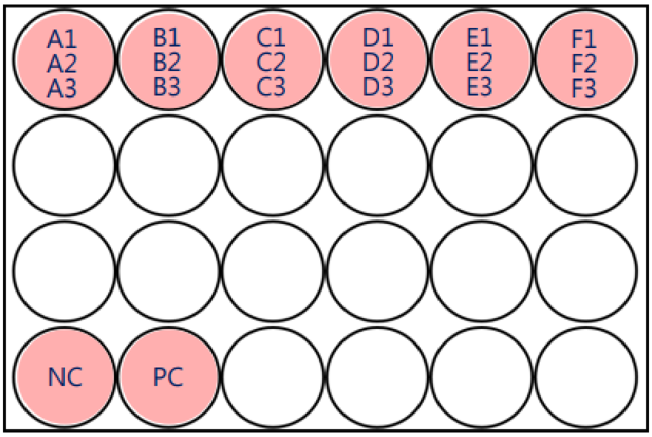

Figure S2. Diagram of the extraction process for the cytotoxicity test (indirect method)

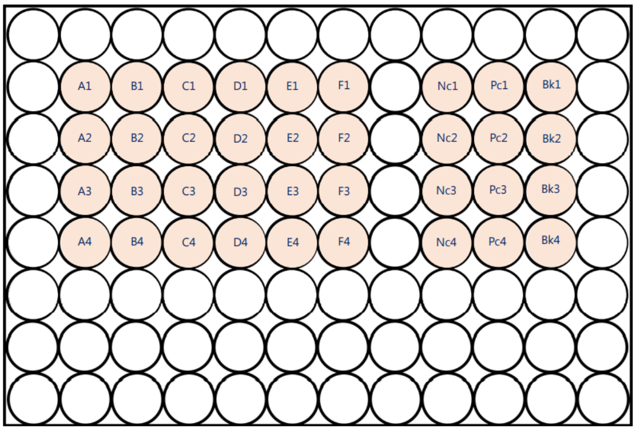

Figure S3. Diagram illustrating the Cell Counting Kit-8 assay procedure for the cytotoxicity test (indirect method).

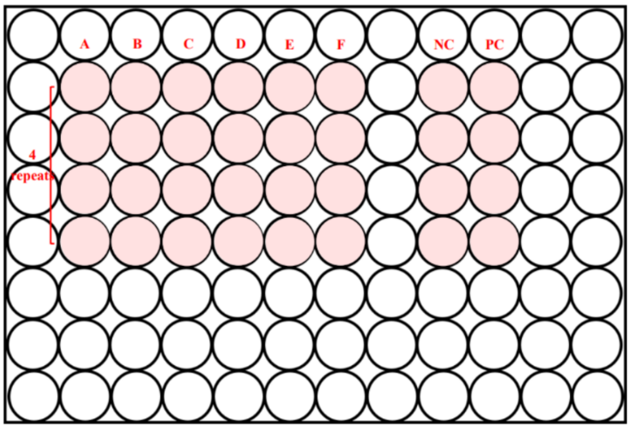

Figure S4. Diagram illustrating the seeding of cells for the cytotoxicity test (indirect method)

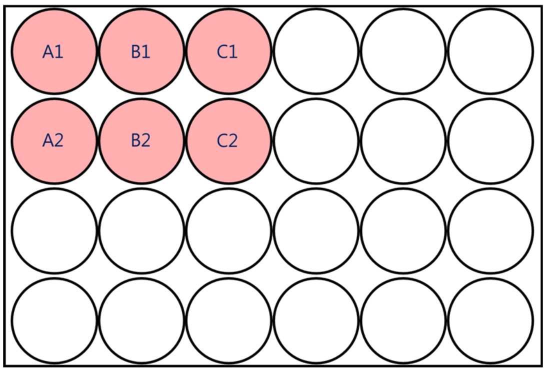

Figure S5. Diagram illustrating the seeding of cells for the direct cell osteogenic differentiation test

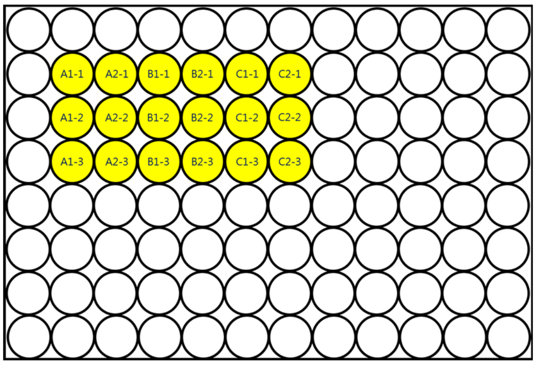

Figure S6. Diagram illustrating the ALP Assay procedure for the direct cell osteogenic differentiation test.

Table S1. Hydrophilicity Test result. Weight change before and after soaking the membrane in hyaluronic acid

	**Membrane thickness (mm)**	**Before soaking (g)**	**After soaking (g)**	**ΔWeight (g)**	**ΔWeight (%)**
Sample 1	0.322	0.0707	0.0897	0.0190	26.9
Sample 2	0.316	0.0707	0.0879	0.0172	24.3
Sample 3	0.321	0.0706	0.0883	0.0177	25.1

Table S2. Mechanical strength results were obtained for electrospun membranes with different ratios of calcium phosphate (β-TCP) powder at a stretching speed of 12.5 mm/min

PLA+10% β-TCP					
	Force(N)	Thickness(mm)	Width(mm)	Stress(Mpa)(N/mm^2)	Strain(%)
Sample 1	0.3929933	0.149	3.52	0.749	30.429
Sample 2	0.3987153	0.161	3.18	0.779	29.448
Sample 3	0.3623168	0.15	2.84	0.851	28.618
Average	0.384675133	0.153	3.18	0.793	29.499
PLA+20% β-TCP					
	Force(N)	Thickness(mm)	Width(mm)	Stress(Mpa)(N/mm^2)	Strain(%)
Sample 1	0.4051526	0.158	3.18	0.806	22.869
Sample 2	0.4008611	0.164	3.18	0.769	22.861
Sample 3	0.4252593	0.172	2.81	0.880	21.051
Average	0.410424333	0.165	3.06	0.818	22.261
PLA+30% β-TCP					
	Force(N)	Thickness(mm)	Width(mm)	Stress(Mpa)(N/mm^2)	Strain(%)
Sample 1	0.2444585	0.095	3.18	0.809	8.321
Sample 2	0.2150536	0.091	2.63	0.899	9.233
Sample 3	0.2348423	0.094	2.87	0.870	6.205
Average	0.231451467	0.093	2.89	0.859	7.920
PLA+40% β-TCP					
	Force(N)	Thickness(mm)	Width(mm)	Stress(Mpa)(N/mm^2)	Strain(%)
Sample 1	0.7530848	0.104	2.99	2.422	2.031
Sample 2	0.7359982	0.105	3.12	2.247	1.783
Sample 3	0.6736119	0.107	2.28	2.761	1.640
Average	0.7208983	0.105	2.80	2.477	1.818

Table S1. Mechanical strength results were obtained for electrospun membranes with different textures at a stretching speed of 1 mm/min

The membrane texture is loose and soft					
	Force(N)	Thickness(mm)	Width(mm)	Stress(Mpa)(N/mm^2)	Strain(%)
Sample1	0.971874	0.358	3.18	0.854	9.498
Sample2	0.613531	0.271	3.18	0.712	11.229
Sample3	0.689666	0.28	3.18	0.775	13.888
Average	0.758357	0.303	3.18	0.780	11.538
The membrane texture is firm					
	Force(N)	Thickness(mm)	Width(mm)	Stress(Mpa)(N/mm^2)	Strain(%)
Sample1	1.182953	0.252	3.18	1.476	22.822
Sample2	0.8273919	0.238	3.18	1.093	19.346
Sample3	0.4797777	0.18	3.18	0.838	25.427
Average	0.830040867	0.223	3.18	1.136	22.532
The membrane texture is firm					
	Force(N)	Thickness(mm)	Width(mm)	Stress(Mpa)(N/mm^2)	Strain(%)
Sample1	1.019637	0.248	3.18	1.293	22.628
Sample2	0.8560022	0.255	3.18	1.056	27.300
Sample3	0.7354419	0.23	3.18	1.006	24.307
Average	0.870360367	0.244	3.18	1.118	24.745
